# Transactions of the Med. Society of the State of Pennsylvania for 1854

**Published:** 1854-12

**Authors:** 


*Transactions of the Medical Society of the State of Pennsylvania for* 1854.—We have received the above through Messrs. Miller, Orton & Mulligan. As usual the Pennsylvania Society has issued a very creditable volume. The system, commenced in previous years, of county reports on medical topography, with maps, is continued, and as it progresses toward completion it acquires greater value.

Most of the present volume is made up from reports of county societies, and all of them have an interest and value which is not merely local. We know of no state society which accomplishes more than Pennsylvania. In our own state the society has suffered from the general decline of the county societies, owing to imprudent and meddlesome legislation. As these are again reörganized, we may hope that the Transactions of the New York State Society may correspond somewhat to the importance of the great state over which it has jurisdiction. H.

